# Severe Eosinophilic Asthma: From Immunopathology to Pharmacological Treatment

**DOI:** 10.3390/jcm15103845

**Published:** 2026-05-16

**Authors:** Daniela Pastore, Chiara Lupia, Emanuela Chiarella, Giovanna Lucia Piazzetta, Giuseppe Mazza, Giuseppe Neri, Albino Petrone, Andrea Bruni, Federico Longhini, Eugenio Garofalo, Girolamo Pelaia, Corrado Pelaia

**Affiliations:** 1 Department of Health Sciences, University “Magna Graecia” of Catanzaro, 88100 Catanzaro, Italy; danielapastore11@gmail.com (D.P.); chiaralupia1996@gmail.com (C.L.); pelaia@unicz.it (G.P.); 2 Department of Medical and Surgical Sciences, University “Magna Graecia” of Catanzaro, 88100 Catanzaro, Italy; emanuelachiarella@unicz.it (E.C.); giovannapiazzetta@hotmail.it (G.L.P.); giuseppe.mazza@unicz.it (G.M.); giuseppeneri91@gmail.com (G.N.); flonghini@unicz.it (F.L.); eugenio.garofalo@unicz.it (E.G.); 3 Department of Respiratory Diseases, Annunziata Hospital, 87100 Cosenza, Italy; alb.petronedoc@gmail.com; 4 Department of Pharmacy, Health and Nutritional Sciences, University of Calabria, 87036 Rende, Italy; andrea.bruni@unical.it

**Keywords:** severe asthma, eosinophil, immunopathology, biologic therapy

## Abstract

**Background:** Asthma is a heterogeneous chronic airway disease characterized by inflammation, airflow obstruction, hyperresponsiveness, and remodeling. Severe eosinophilic asthma is driven by eosinophilic inflammation, which contributes to tissue damage, recurrent exacerbations, and progressive impairment of airway structure and function. Eosinophils play a central role through the release of cytokines, cytotoxic granule proteins, and extracellular traps, and their persistence in the airways is sustained by type 2 inflammatory pathways, particularly interleukin-5-mediated signaling. A better understanding of eosinophil biology has promoted the development of targeted therapies, including anti-interleukin-5/interleukin-5 receptor agents and biologics that indirectly modulate eosinophilic inflammation, such as anti-interleukin-4 receptor alpha and anti-thymic stromal lymphopoietin antibodies. **Aim:** This narrative review summarizes the immunopathology of eosinophilic asthma and links eosinophil biology to current and emerging pharmacological strategies. We discuss biologics that directly target the IL-5/IL-5 receptor axis, as well as agents that indirectly modulate eosinophilic inflammation, including IL-4 receptor alpha and TSLP blockade. We also review the clinical positioning of available biologics, focusing on blood eosinophils, FeNO, exacerbation history, oral corticosteroid exposure, lung function, type 2 comorbidities, treatment response, remission and switching. **Conclusions:** Overall, eosinophilic inflammation remains a central therapeutic target and a key component of precision medicine in severe asthma, but biologic selection should be individualized and reassessed through multidomain clinical outcomes.

## 1. Introduction

Asthma is a chronic respiratory disease characterized by symptoms such as shortness of breath, wheezing, chest tightness, and cough, together with airway inflammation, bronchoconstriction, hyperresponsiveness, and remodeling [1]. Asthma pathogenesis involves a complex interaction between cellular and molecular elements, including inflammatory mediators and cytoskeletal proteins [2]. In particular, severe asthma is known to encompass multiple phenotypes and endotypes, which differ in clinical presentation, underlying pathogenetic pathways, and treatment response [1]. Severe asthma comprises multiple clinical phenotypes and inflammatory endotypes, broadly including type 2-high allergic and non-allergic eosinophilic disease, as well as type 2-low neutrophilic and paucigranulocytic asthma [3]. Patients with severe asthma often report poor control of respiratory symptoms despite the use of inhaled corticosteroids (ICS) and long-acting β_2_ agonists (LABA). When needed, these medications are supplemented with additional therapies such as oral corticosteroids (OCS), leukotriene modifiers, long-acting muscarinic antagonists (LAMA), and targeted biologic molecules [1,4]. Severe asthma is also characterized by frequent exacerbations, defined as increasing symptoms linked to airflow restriction that require treatment intensification or hospitalization [5]. Regardless of their allergy status, many individuals with severe asthma have significant eosinophilic airway inflammation. Indeed, the International Severe Asthma Registry revealed that more than 80% of individuals with the most severe clinical characteristics may have eosinophilic asthma [6,7]. In type 2 (T2) asthma, group 2 innate lymphoid cells (ILC2) and T helper 2 (Th2) lymphocytes are involved in the development of airway eosinophilia, which is dependent on pathological networks involving close interactions between innate and adaptive immunity. These cells produce a number of cytokines, including interleukin-5 (IL-5), interleukin-4 (IL-4), and interleukin-13 (IL-13). The primary regulator of eosinophil biology, including differentiation, maturation, survival, and activation, is IL-5 [8]. In a normal state, eosinophils die and are removed by macrophages without producing inflammation. However, impairment of this apoptotic clearance mechanism may prolong eosinophil persistence in the airways, thereby worsening eosinophilic inflammation and asthma severity [9,10].

Although several reviews have addressed biologic therapies in severe asthma, the present narrative review aims to provide an integrated eosinophil-centered perspective, linking eosinophil immunopathology with pharmacological targeting and clinical decision-making. Specifically, this review focuses on how eosinophil biology informs the positioning of anti-IL-5/IL-5R agents and biologics that indirectly modulate eosinophilic inflammation, how biomarkers and type 2 comorbidities can guide treatment selection, and how multidomain response and remission concepts may influence monitoring and switching strategies. Therefore, the distinctive contribution of this review is the integration of mechanistic immunology, current and emerging pharmacological options, and practical clinical positioning within a precision-medicine framework for severe eosinophilic asthma.

## 2. Methods: Narrative Literature Search Strategy

This manuscript was designed as a narrative review. A structured literature search was performed in PubMed/MEDLINE, Scopus, Web of Science, and Google Scholar to identify relevant articles on severe eosinophilic asthma, eosinophil biology, and biologic therapies. The search covered publications from January 2010 to April 2026, although older seminal articles were also included when considered essential for the mechanistic or historical context. Search terms included combinations of: “severe asthma”, “eosinophilic asthma”, “eosinophils”, “type 2 inflammation”, “IL-5”, “IL-5 receptor”, “mepolizumab”, “reslizumab”, “benralizumab”, “depemokimab”, “dupilumab”, “tezepelumab”, “TSLP”, “FeNO”, “biologics”, “switching”, “clinical response”, “clinical remission”, and “oral corticosteroids”. Eligible sources included randomized controlled trials, extension studies, real-world and registry studies, systematic and narrative reviews, international guidelines, and relevant mechanistic studies. Articles were prioritized according to clinical relevance, methodological quality, recency, and contribution to the understanding of eosinophilic inflammation, treatment selection, response assessment, switching, and remission. Studies not focused on severe asthma, eosinophilic inflammation, biologic therapy, or clinically relevant mechanistic pathways were excluded. Because this was a narrative review, no formal meta-analysis, PRISMA flow diagram, or risk-of-bias assessment was performed. To reduce potential selection bias, we aimed to include evidence from different research groups, international guidelines, pivotal trials, and multicenter real-world cohorts.

## 3. The Role of Eosinophils in Lung Inflammation

Eosinophils are specialized granulocytes that play key roles in several inflammatory responses. Eosinophil development begins in the bone marrow, where hematopoietic stem and progenitor cells differentiate into eosinophil/mast cell progenitors. Eosinophils may also derive from CD34+ eosinophil progenitors (EoPs), which are present outside of bone marrow, blood, and especially lung tissue [11]. Notably, eosinophil progenitor cells may persist during anti-IL-5 therapy, suggesting that in situ eosinophilopoiesis can contribute to chronic airway eosinophilia [12]. Although new research suggests that eosinophils may have a variety of homeostatic and regulatory roles, they have historically been categorized as effector cells with widespread cytotoxic activity [13]. Cytokines such as interleukin-3 (IL-3), IL-5, and granulocyte-macrophage colony-stimulating factor (GM-CSF) stimulate the development and maturation of eosinophils from CD34+ hematopoietic progenitors in the bone marrow [14]. Whereas GM-CSF and IL-3 are less selective and also promote the growth of other leukocytes, such as neutrophils and macrophages, IL-5 acts more selectively on eosinophils and basophils [15]. Two main eosinophil populations can be distinguished: homeostatic eosinophils and inflammatory eosinophils. These two populations differ mainly in their development and recruitment: inflammatory eosinophils require IL-5 for differentiation from precursor cells and subsequent migration to the lungs, whereas homeostatic eosinophils differentiate in the bone marrow in a relatively IL-5-independent manner [16]. Additionally, eosinophil differentiation was prolonged as long as CD34+ progenitor cells were stimulated with IL-3, IL-5, and GM-CSF since this led to an overexpression of the IL-5 receptor (IL-5R) in these stem cells [17]. Complex surface receptor structures seen on mature eosinophils interact with activating, inhibitory, and chemokine factors. Eosinophils are drawn to inflammatory areas by chemokines such as eotaxin-1, -2, and -3, RANTES, and prostaglandin D2 (PGD2) via the receptors chemokine receptor 3 (CCR3), chemokine receptor 1 (CCR1), and prostaglandin D2 receptor 2 (DP2), also known as chemoattractant receptor-homologous molecule expressed on Th2 cells (CRTH2). GM-CSF, IL-5, and IL-3 act as activating signals by binding to their respective receptors, namely GM-CSFR, IL-5R, and IL-3R. Inhibitory pathways in eosinophils also involve receptors such as Siglec-8 and regulatory signals related to TGF-β [18]. Both mature and immature eosinophils may leave the bone marrow after an inflammatory reaction and enter distant tissues in 8–24 h, usually staying there for 3–8 days [19]. Eosinophil trafficking to inflamed tissues depends on coordinated rolling, adhesion, and transmigration mediated by selectins, integrins, and chemokine receptors. Circulating eosinophils express L-selectin and P-selectin glycoprotein ligand-1, whereas endothelial selectins contribute to the rolling step. Firm adhesion and transendothelial migration are mainly mediated by integrins such as VLA-4 and Mac-1 through interactions with VCAM-1 and ICAM-1, respectively. In parallel, CCR3 engagement by eotaxins promotes eosinophil recruitment and activation [20,21,22,23,24,25,26]. Eosinophils contribute to airway remodeling and airway hyperresponsiveness (AHR), promoting goblet cell hyperplasia, smooth muscle hypertrophy, and extracellular matrix deposition, which may lead to airway wall thickening and fibrosis [27].

Eosinophil-mediated tissue injury is largely related to the release of granule-derived mediators. Eosinophils contain both specific granules and immature specific granules. Specific granules include a wide range of mediators involved in tissue inflammation and injury, including growth factors, cytokines, chemokines, basic proteins, and enzymes. Major constituents include eosinophil-derived neurotoxin (EDN), eosinophil peroxidase (EPX), major basic proteins (MBP-1 and MBP-2), and eosinophil cationic protein (ECP). In contrast to these specific granule mediators, Charcot-Leyden crystal (CLC) protein is mainly found in immature specific granules, also known as primary granules [28,29]. Eosinophils can release granule contents through different mechanisms. Classical exocytosis involves direct fusion of granules with the plasma membrane. Composite exocytosis occurs when granules fuse intracellularly before releasing their contents extracellularly. Piecemeal degranulation (PMD), the most common mechanism, involves vesicular transport of selected granule contents to the cell membrane. These vesicles are often referred to as “sombrero vesicles” because of their characteristic morphology. Finally, cytolysis is a non-apoptotic form of cell death characterized by disruption of the nuclear and plasma membranes, extracellular DNA release, and deposition of intact granules in the extracellular space [30,31].

Activated eosinophils can also release extracellular traps composed of DNA and granule proteins, thereby contributing to host defense and tissue injury [32,33].

## 4. Biologic Treatments Against Eosinophils in Severe Asthma

Due to their significant involvement in the pathobiology of asthma, eosinophils are important biologic targets for the treatment of severe eosinophilic asthma. Currently available therapies that directly target eosinophilic inflammation include anti-IL-5 and anti-IL-5 receptor monoclonal antibodies; other biologics, such as anti-IL-4Rα and anti-thymic stromal lymphopoietin (TSLP) agents, may indirectly reduce eosinophilic inflammation. According to GINA step 5 recommendations, IL-5 blockade and IL-5 receptor antagonism represent effective add-on options for selected patients with severe eosinophilic asthma [34].

### 4.1. Mepolizumab

The first humanized monoclonal antibody approved for treating severe eosinophilic asthma was mepolizumab. It specifically targets IL-5, which regulates eosinophil survival, differentiation, and proliferation. Its activity has been demonstrated both in the blood and in the airways, reducing eosinophil levels in both compartments [35]. Patients with blood eosinophil counts greater than 150 cells/µL at treatment initiation or greater than 300 cells/µL during the previous year, particularly those with at least two exacerbations requiring oral corticosteroids in the preceding year, may benefit from this treatment [36,37]. Mepolizumab is approved for use in patients with severe eosinophilic asthma, with age eligibility depending on the regulatory jurisdiction. Mepolizumab is administered by a subcutaneous injection at a fixed dose of 100 mg every 28 days. In addition to severe eosinophilic asthma, mepolizumab is also indicated for other eosinophil-driven conditions, including hypereosinophilic syndrome (HES), eosinophilic granulomatosis with polyangiitis (EGPA), and chronic rhinosinusitis with nasal polyps (CRSwNP) [38]. Recently, a phase 3, double-blind, randomized, placebo-controlled trial demonstrated that, in patients with eosinophilic phenotype chronic obstructive pulmonary disease (COPD), mepolizumab added to triple inhaled therapy reduced the annualized risk of moderate or severe exacerbations [39]. Several studies have evaluated the effectiveness of mepolizumab in uncontrolled severe eosinophilic asthma, showing favorable effects on symptom control, exacerbation rate, maintenance OCS dose, number of hospitalizations, and lung function [40,41]. According to the recent multicenter MESILICO study, patients with late-onset severe asthma characterized by eosinophilic inflammation and impaired reversibility showed both significant clinical improvement and a reduction in airway remodeling indices after one year of mepolizumab treatment [42].

### 4.2. Reslizumab

Reslizumab is a humanized IgG4 monoclonal antibody that reduces blood and airway eosinophils by binding to IL-5. It is generally considered for patients with blood eosinophil counts ≥ 400 cells/µL and a history of asthma exacerbations during the previous 12 months [43]. Reslizumab has been approved for adults with severe eosinophilic asthma that remains uncontrolled despite high-dose inhaled corticosteroids and another controller medication. Reslizumab was able to significantly increase forced expiratory volume in 1 s (FEV_1_) and decrease blood and sputum eosinophil counts, according to preliminary phase 2 research [44]. After that, two more phase 3 trials were carried out in which reslizumab was given to participants with severe asthma whose blood eosinophil counts were greater than 400 cells/μL. These patients experienced a reduction in asthma exacerbation rates of more than 50%, together with improved asthma control and a significant increase in FEV1 [45]. Moreover, reslizumab has been shown to enhance lung function by improving forced expiratory flow at 25–75% of forced vital capacity (FEF_25–75_) in addition to increasing FEV_1_ [46]. This medication is administered at a dose of 3 mg/kg every 4 weeks. In open-label and observational trials, reslizumab has also been shown to be useful in treating EGPA; however, this benefit was only seen in a limited patient cohort [47].

### 4.3. Benralizumab

The humanized monoclonal antibody benralizumab (IgG1κ) binds particularly strongly to the α subunit of the IL-5 receptor, expressed by basophils and eosinophils, preventing it from interacting with IL-5. In addition, benralizumab induces antibody-dependent cell-mediated cytotoxicity (ADCC), mediated by activated natural killer cells, leading to eosinophil depletion. Benralizumab also encourages macrophages to carry out antibody-dependent cellular phagocytosis (ADCP), whereby eosinophils are eliminated via phagocytosis and efferocytosis. Benralizumab-activated NK cells produce stimulatory cytokines such as interferon-γ (IFN-γ) to promote macrophage cytotoxicity via TNF-α, which may start TNF receptor 1 (TNFR1)-dependent eosinophil apoptosis [48,49]. This biologic drug may be used as an add-on treatment for patients with uncontrolled severe eosinophilic asthma who have a blood eosinophil count of at least 300 cells/µL [50]. It is administered subcutaneously at a dose of 30 mg every 4 weeks for the first three doses and every 8 weeks thereafter. In severe uncontrolled asthma patients with an eosinophilic phenotype, the SIROCCO and CALIMA trials showed that subcutaneous benralizumab, administered at 30 mg every 4 weeks or every 8 weeks after the first three doses, significantly reduced exacerbation rates and improved lung function and asthma symptom scores compared with placebo [51,52]. When compared to a placebo, benralizumab showed a clinically significant advantage in lowering the use of OCS and exacerbations in the phase 3 ZONDA research [53]. The long-term BORA and MELTEMI extension studies confirmed the safety and efficacy of benralizumab for up to two and five years of continuous treatment, respectively [54,55]. Greater clinical benefit has been observed in adults, patients with nasal polyposis, impaired lung function, and those with more than three exacerbations in the preceding 12 months. Significant improvements have also been reported in patient-reported outcomes, including the Asthma Control Questionnaire (ACQ), Asthma Control Test (ACT), and Asthma Quality of Life Questionnaire (AQLQ) [56,57].

### 4.4. Depemokimab

Depemokimab, a long-acting human monoclonal antibody targeting IL-5, represents an emerging anti-eosinophilic strategy designed to provide sustained eosinophil suppression with less frequent dosing compared with currently available anti-IL-5 biologics. In phase 3 trials involving patients with severe asthma and type 2 inflammation, depemokimab significantly reduced exacerbation rates, supporting its potential role as a convenient long-interval biologic option for selected patients with severe eosinophilic asthma [58,59].

## 5. Existing Biologic Treatments That Indirectly Target Eosinophils

### 5.1. Dupilumab

Beyond biologic approaches that directly target eosinophil survival or depletion, additional agents can modulate eosinophil-driven pro-inflammatory and pro-remodeling pathways, thereby acting as indirect anti-eosinophil therapies. Dupilumab, a completely human monoclonal antibody that was approved in 2018, recognizes and inhibits the α-subunit of the IL-4 receptor, which is necessary for the activation of signaling pathways that are activated by both IL-4 and IL-13. IL-13 contributes to asthma pathobiology by promoting mucus production, airway remodeling, and bronchial hyperresponsiveness. IL-4 promotes eosinophil transmigration across the vascular endothelium and also stimulates Th2 cell differentiation and B-cell class switching to IgE [60]. Dupilumab has been approved as add-on maintenance therapy for patients with moderate-to-severe asthma and evidence of type 2 inflammation, as indicated by elevated fractional exhaled nitric oxide (FeNO) and/or blood eosinophils. Dupilumab is administered subcutaneously either as a loading dose of 600 mg, followed by 300 mg every 2 weeks, or as a loading dose of 400 mg, followed by 200 mg every 2 weeks. It was initially approved for atopic dermatitis and was subsequently authorized for severe asthma. It is also approved for other type 2 inflammatory diseases, including chronic rhinosinusitis with nasal polyps and eosinophilic esophagitis, and in some settings for eosinophilic COPD [61].

### 5.2. Tezepelumab

Tezepelumab is a fully human anti-TSLP monoclonal IgG2λ antibody, approved as an add-on maintenance treatment for severe asthma without phenotypic or biomarker restrictions [62]. It is directed against TSLP, an important epithelial alarmin. TSLP influences both T2 and non-T2, or T2-low, asthma and is released by airway epithelial cells in response to various environmental and inflammatory stimuli [63]. TSLP has also been implicated in neutrophilic inflammation through dendritic cell activation and promotion of Th17 polarization [64]. Furthermore, TSLP is a potent direct activator of ILC2, which reacts to this alarmin by extending their lifespan, generating the type 2 cytokines listed above, and displaying resistance to corticosteroids [65]. Several studies demonstrated the efficacy of this biologic drug. In particular, the NAVIGATOR study, a phase 3 randomized controlled trial, showed a marked reduction in exacerbation rates, particularly in patients with higher blood eosinophil counts and FeNO levels [66]. The PATHWAY trial was the first clinical study to show that tezepelumab was effective in treating people with severe, uncontrolled asthma [67]. In the phase 3 SOURCE study, tezepelumab did not significantly improve oral corticosteroid dose reduction versus placebo in the overall population, although signals of benefit were observed in selected analyses and patient subsets [68]. The UPSTREAM research revealed that inhibiting TSLP may have advantages beyond lowering type 2 airway inflammation, as shown by a decrease in airway hyperresponsiveness to mannitol [69]. Finally, the DESTINATION extension study supported the long-term safety and efficacy of tezepelumab over up to 104 weeks [70].

A practical clinical positioning of currently available biologics in severe eosinophilic asthma is represented in Table 1.

## 6. Possible Future Treatments Against Eosinophils

Pro-apoptotic antibodies, prostanoid receptor antagonists, chemokine receptor antagonists, and kinase or transcription factor inhibitors are among the investigational anti-eosinophil strategies currently being evaluated in preclinical and clinical studies.

### 6.1. JAK Inhibitors and GATA-3-Targeted Approaches

The Janus kinase/signal transducer and activator of transcription (JAK/STAT) pathway is involved in signaling mediated by pro-inflammatory type 2 cytokines [71,72]. Therefore, experimental therapies targeting this pathway are being investigated as additional strategies to modulate eosinophilic inflammation in severe asthma. In murine models of allergic airway inflammation, CP-690,550 (tofacitinib) reduced bronchoalveolar eosinophilia, supporting JAK/STAT signaling as a potential therapeutic target in eosinophilic asthma [73,74]. In fact, GATA-3 increases the production of type 2 cytokines, particularly IL-5, by encouraging naïve Th0 cells to commit to the Th2 lineage [75,76]. In recent years, GATA-3-targeting DNAzymes have been developed. According to a placebo-controlled study, the inhaled GATA-3-specific DNAzyme SB010 effectively reduced allergen-induced sputum eosinophilia and plasma levels of IL-5 in atopic asthmatic patients [77].

### 6.2. Siglec-8

Siglec-8 belongs to the sialic acid-binding immunoglobulin-like lectin family and is expressed by eosinophils, mast cells, and basophils [78,79]. Binding of the monoclonal antibody lirentelimab/AK002 to Siglec-8 can induce antibody-dependent cellular cytotoxicity, leading to eosinophil apoptosis and reduced sputum eosinophil counts in patients with asthma [80]. Therefore, antibody-mediated induction of eosinophil apoptosis may represent a potential therapeutic strategy for type 2 asthma and other eosinophil-associated diseases [81].

### 6.3. CRTH2 Inhibitors

The prostaglandin D2 receptor CRTH2 is expressed by several immune cells, including blood eosinophils [82]. A number of oral CRTH2 antagonists have been tested to assess their possible therapeutic benefits on airway eosinophilic inflammation [83]. Early-stage studies in patients with asthma have shown the potential of fevipiprant, an oral, reversible competitive antagonist of CRTH2. In particular these studies have shown a reduction in sputum eosinophils and an improvement in lung function and quality of life [84]. Subsequently, two phase-3 trials, LUSTER-1 and LUSTER-2, failed to meet the primary endpoint, which is represented by the reduction in the number of exacerbations [85]. In individuals with mild persistent asthma, the CRTH2 antagonist OC000459, known as timapiprant, significantly increased FEV_1_ and lowered sputum eosinophil levels when compared to a placebo [86].

### 6.4. CCR3 Antagonists

The chemokine receptor CCR3 has been identified as an eosinophil migratory driver [87]. Patients with mild-to-moderate atopic asthma who received the selective oral CCR3 antagonist AXP1275 prior to an inhaled allergen challenge showed a significant improvement in bronchial hyperresponsiveness to methacholine and a non-significant trend towards a decrease in antigen-dependent airway eosinophilia [88]. GW766994, another oral CCR3 antagonist, had comparable outcomes [89]. However, these oral CCR3-targeted approaches have not reached the same clinical status as approved biologics, because the available evidence remains limited to early-phase studies and has shown only partial effects on eosinophilic inflammation. In particular, the reduction in airway eosinophilia with AXP1275 was only a non-significant trend, suggesting that more robust and reproducible clinical evidence is needed before these agents can be considered for routine use.

TPI ASM8, a combination of two antisense oligonucleotides, targets CCR3 and the common β-chain shared by the receptors for IL-5, IL-3, and GM-CSF. CCR3 binds several chemokines involved in eosinophil recruitment, including eotaxin (CCL11), eotaxin-3 (CCL26), MCP-3 (CCL7), MCP-4 (CCL13), and RANTES [90]. TPI ASM8 substantially decreased early and late asthmatic responses and allergen-induced airway eosinophilia in individuals with moderate allergic asthma by 46% when compared to a placebo [90]. In addition, TPI ASM8 inhibited the allergen-induced increase in airway eosinophil cationic protein concentrations in a dose-dependent manner [91].

## 7. Clinical Positioning of Biologic Therapies in Severe Eosinophilic Asthma

From a clinical perspective, the availability of multiple biologics means that treatment selection in severe eosinophilic asthma should be based on an integrated assessment of exacerbation history, maintenance oral corticosteroid (OCS) requirement, blood eosinophil count, fractional exhaled nitric oxide (FeNO), lung function, age at disease onset, and type 2 comorbidities, rather than on a single biomarker alone [92]. In patients with a clearly eosinophilic phenotype, especially those with recurrent exacerbations, OCS dependence, late-onset asthma, and chronic rhinosinusitis with nasal polyps, anti-IL-5/IL-5R agents are often the most rational first choice because they directly target the effector pathway driving eosinophil survival and tissue accumulation. Dupilumab may be particularly suitable when eosinophilic inflammation coexists with elevated FeNO and clinically relevant comorbidities such as nasal polyposis or atopic dermatitis, whereas tezepelumab may represent a valuable option when type 2 biomarkers are overlapping, discordant, or partially suppressed by corticosteroid exposure, and when a broader upstream blockade is clinically desirable [93]. Thus, in routine practice, biologic selection should be phenotype-driven and individualized, while also taking into account administration schedule, previous biologic exposure, patient preference, and local access criteria [94].

Clinical management does not end with biologic initiation. Treatment response should be evaluated multidimensionally by considering exacerbation rate, symptom control, lung function, reduction in OCS burden, and the course of associated comorbidities, because improvement in a single domain may not fully reflect overall benefit [95]. In patients with persistent exacerbations, ongoing OCS dependence, inadequate symptom control, or persistent type 2 inflammation despite treatment, clinicians should first reassess adherence, inhaler technique, alternative diagnoses, and treatable traits before considering a switch to another biologic [96]. Real-world studies indicate that switching is most frequently required in subjects with suboptimal control despite biologic therapy, particularly when high blood eosinophils, elevated FeNO, or nasal polyposis persist [97].

The concept of remission in severe asthma should be interpreted cautiously because definitions vary across studies [98]. Clinical remission generally refers to the absence of exacerbations, good symptom control, stable or improved lung function and no maintenance OCS use, although the exact combination of these domains differs among studies [99]. Complete remission is a more stringent concept and may additionally require suppression of underlying inflammatory activity, including biomarker normalization. Multidomain remission refers to the simultaneous achievement of predefined clinical, functional and treatment-related targets. Therefore, clinical remission should not be used interchangeably with reduction in exacerbations, symptomatic improvement, OCS sparing, or biomarker reduction alone [100]. The achievement of clinical remission appears more likely in patients with lower baseline impairment, shorter asthma duration, fewer exacerbations, better lung function and lower long-term OCS exposure at the time biologic therapy is started [98]. Accordingly, biologic therapy in severe eosinophilic asthma should be regarded as a dynamic precision-medicine strategy, in which the initial choice and any subsequent switch are guided by the evolving clinical phenotype and by multidomain treatment response.

### Proposed Clinical Framework

In clinical practice, biologic selection should follow a stepwise approach: first, confirmation of severe asthma and optimization of adherence, inhaler technique and comorbidities; second, identification of type 2 inflammation through blood eosinophils, FeNO, exacerbation history, OCS exposure and comorbid type 2 diseases; third, selection of the biologic according to the dominant clinical and inflammatory profile; fourth, reassessment of response using multidomain outcomes; and fifth, consideration of switching when response remains inadequate after treatable traits have been reassessed.

## 8. Conclusions

**Figure 1:**
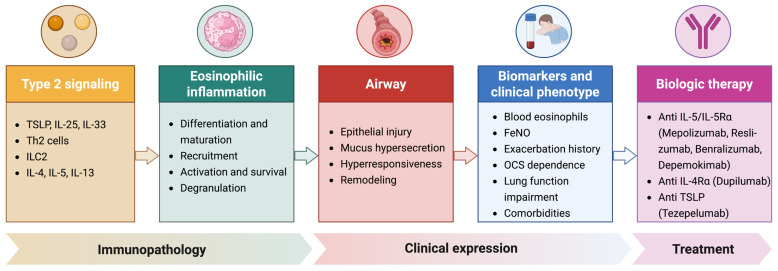
Schematic overview of the pathogenesis of severe eosinophilic asthma and its therapeutic targets. Airway epithelial activation induces type 2 inflammation (IL-4, IL-5, IL-13), leading to eosinophil recruitment, activation, and airway damage. Current biologic therapies targeting IL-5/IL-5R, IL-4Rα, and TSLP are also illustrated. Created in BioRender. Pelaia, C. (2026) https://BioRender.com/crkaat3 (accessed on 4 May 2026).

Eosinophilic inflammation is one of the major drivers of exacerbation risk, persistent airway inflammation, increased disease severity, and reduced responsiveness to conventional anti-inflammatory treatment in a substantial proportion of patients with severe asthma. Over the past decade, a deeper understanding of eosinophil biology has led to the development of targeted therapies that have significantly improved disease control, reduced exacerbation frequency, and decreased oral corticosteroid exposure in many patients (Figure 1). However, currently available biologics do not completely overcome the heterogeneity of severe asthma, and treatment response remains variable across individuals. In this context, the management of severe eosinophilic asthma should not rely solely on mechanistic or biomarker-based assumptions, but rather on an integrated clinical approach that includes exacerbation history, blood eosinophil count, FeNO, comorbid type 2 diseases, oral corticosteroid burden, lung function, and patient-related factors. Therefore, biologic therapy should be regarded as part of a dynamic precision-medicine strategy, in which treatment selection, monitoring of multidomain response, and switching decisions are tailored to the evolving clinical phenotype. Future studies should further refine predictive biomarkers, clarify the optimal positioning of available biologics, and better define remission targets and long-term disease modification in order to achieve a more personalized and clinically effective management of severe eosinophilic asthma.

## Figures and Tables

**Table 1:** Practical clinical positioning of currently available biologics in severe eosinophilic asthma.

Biologic	Target	Main Clinical Phenotype/Ideal Candidate	Key Biomarkers/Clinical Clues	Relevant Comorbidities	Main Clinical Advantages	Potential Limitations/Practical Caveats	Route and Schedule
Mepolizumab	IL-5	Classic severe eosinophilic asthma, especially in patients with recurrent exacerbations and/or maintenance OCS exposure	Blood eosinophils ≥ 150 cells/µL at treatment initiation or ≥300 cells/µL in the previous year; recurrent exacerbations	CRSwNP, EGPA, HES	Direct anti-eosinophil effect; reduction in exacerbations; OCS-sparing effect; broad real-world evidence	Biomarker-driven selection is important; response may be less evident when eosinophilia is weak or suppressed by corticosteroids	Subcutaneous; 100 mg every 4 weeks
Reslizumab	IL-5	Severe eosinophilic asthma in adults with marked eosinophilia and recurrent exacerbations	Blood eosinophils generally ≥400 cells/µL; exacerbation history; improvement in FEV1 may be expected in responders	EGPA (limited evidence)	Direct anti-eosinophil effect; favorable impact on exacerbations and lung function	Use is limited to adults; intravenous administration may be less practical in routine care	Intravenous; 3 mg/kg every 4 weeks
Benralizumab	IL-5 receptor α	Severe eosinophilic asthma with frequent exacerbations, OCS dependence, adult-onset disease, and/or nasal polyposis	Blood eosinophils ≥ 300 cells/µL; recurrent exacerbations; maintenance OCS use; late-onset asthma	CRSwNP	Near-complete eosinophil depletion; marked OCS-sparing effect; convenient maintenance interval; strong efficacy in exacerbation reduction	Choice should still be individualized according to phenotype and multidomain response	Subcutaneous; 30 mg every 4 weeks for the first 3 doses, then every 8 weeks
Dupilumab	IL-4 receptor α (blocks IL-4/IL-13 signaling)	Type 2 asthma with eosinophilic inflammation and/or high FeNO, particularly when comorbid type 2 disease is clinically relevant	Elevated FeNO and/or blood eosinophils; overlapping type 2 phenotype	CRSwNP, atopic dermatitis, eosinophilic esophagitis	Useful when upper airway or dermatologic comorbidities influence biologic choice; indirect reduction of eosinophilic inflammation	Not a direct anti-IL-5 strategy; best positioned when broader type 2 blockade is desirable	Subcutaneous; 200 or 300 mg every 2 weeks after loading dose
Tezepelumab	TSLP	Severe asthma with eosinophilic features but also in patients with overlapping, discordant, or partially steroid-suppressed biomarkers	Can be used without phenotypic or biomarker restriction; greater benefit generally seen with higher eosinophils and/or FeNO	Potentially useful when phenotype is less clearly restricted to a single downstream pathway	Upstream blockade; broad positioning in severe asthma; useful when biomarkers are not fully concordant	Broader indication may make patient selection less straightforward; response still needs multidomain reassessment	Subcutaneous; 210 mg every 4 weeks
Depemokimab	IL-5	Severe eosinophilic asthma with recurrent exacerbations, particularly in patients suitable for long-interval biologic administration	Blood eosinophils consistent with type 2/eosinophilic inflammation; exacerbation history	Potentially similar to other anti-IL-5 candidates (to be further defined)	Direct anti-eosinophil effect; prolonged IL-5 blockade; reduced exacerbation risk; highly convenient extended dosing interval	Emerging therapy with more limited long-term real-world experience compared with established biologics	Subcutaneous; every 6 months

## Data Availability

No new data were created or analyzed in this study.

## Abbreviations

ACQ, Asthma Control Questionnaire; ACT, Asthma Control Test; ADCC, antibody-dependent cell-mediated cytotoxicity; ADCP, antibody-dependent cellular phagocytosis; AHR, airway hyperresponsiveness; AQLQ, Asthma Quality of Life Questionnaire; CCR1, chemokine receptor 1; CCR3, chemokine receptor 3; CD15s, Sialyl-Lewis X; CD62L, L-selectin; CD162, P-selectin glycoprotein ligand-1; CLC, Charcot-Leyden crystal; COPD, chronic obstructive pulmonary disease; CRSwNP, chronic rhinosinusitis with nasal polyps; CRTH2, chemoattractant receptor-homologous molecule expressed on Th2 cells; DP2, prostaglandin D2 receptor 2; ECP, eosinophil cationic protein; EDN, eosinophil-derived neurotoxin; EGPA, eosinophilic granulomatosis with polyangiitis; EETs, eosinophil extracellular traps; EoPs, eosinophil progenitors; EPX, eosinophil peroxidase; FeNO, fractional exhaled nitric oxide; FEF_25–75_, forced expiratory flow at 25–75% of forced vital capacity; FEV_1_, forced expiratory volume in 1 s; GM-CSF, granulocyte-macrophage colony-stimulating factor; HES, hypereosinophilic syndrome; ICS, inhaled corticosteroids; IFN-γ, interferon-γ; IgE, immunoglobulin E; IL-3, interleukin-3; IL-4, interleukin-4; IL-5, interleukin-5; IL-5R, interleukin-5 receptor; IL-13, interleukin-13; ILC2, group 2 innate lymphoid cells; JAK, Janus kinase; LABA, long-acting β_2_ agonists; LAMA, long-acting muscarinic antagonists; Mac-1, macrophage-1 antigen; MBP-1, major basic protein-1; MBP-2, major basic protein-2; OCS, oral corticosteroids; PGD2, prostaglandin D2; PMD, piecemeal degranulation; Siglec, sialic acid binding immunoglobulin-like lectin; STAT, signal transducers and activators of transcription; T2, type 2; Th2, T helper 2; TNFR1, tumor necrosis factor receptor 1; TSLP, thymic stromal lymphopoietin; VLA-4, very late antigen-4.

## References

[1] Chung K.F., Wenzel S.E., Brozek J.L., Bush A., Castro M., Sterk P.J., Adcock I.M., Bateman E.D., Bel E.H., Bleecker E.R. (2014). International ERS/ATS guidelines on definition, evaluation and treatment of severe asthma. Eur. Respir. J..

[2] King G.G., James A., Harkness L., Wark P.A.B. (2018). Pathophysiology of severe asthma: We’ve only just started. Respirology.

[3] Papi A., Brightling C., Pedersen S.E., Reddel H.K. (2018). Asthma. Lancet.

[4] Israel E., Reddel H.K. (2017). Severe and Difficult-to-Treat Asthma in Adults. N. Engl. J. Med..

[5] Moore W.C., Bleecker E.R., Curran-Everett D., Erzurum S.C., Ameredes B.T., Bacharier L., Calhoun W.J., Castro M., Chung K.F., Clark M.P. (2007). Characterization of the severe asthma phenotype by the National Heart, Lung, and Blood Institute’s Severe Asthma Research Program. J. Allergy Clin. Immunol..

[6] Kurukulaaratchy R.J., Mistry H. (2021). New Real-World Insights Into Severe Asthma: All About the Eosinophil?. Chest.

[7] Heaney L.G., Perez de Llano L., Al-Ahmad M., Backer V., Busby J., Canonica G.W., Christoff G.C., Cosio B.G., FitzGerald J.M., Heffler E. (2021). Eosinophilic and Noneosinophilic Asthma: An Expert Consensus Framework to Characterize Phenotypes in a Global Real-Life Severe Asthma Cohort. Chest.

[8] D’Amato M., Pastore D., Lupia C., Candia C., Bruni A., Garofalo E., Longhini F., Maglio A., Petrone A., Vatrella A. (2025). Biologic Therapy in Severe Asthma: A Phenotype-Driven and Targeted Approach. J. Clin. Med..

[9] van der Veen T.A., de Groot L.E.S., Melgert B.N. (2020). The different faces of the macrophage in asthma. Curr. Opin. Pulm. Med..

[10] Walsh G.M. (2013). Eosinophil apoptosis and clearance in asthma. J. Cell Death.

[11] Dorman S.C., Efthimiadis A., Babirad I., Watson R.M., Denburg J.A., Hargreave F.E., O'BYrne P.M., Sehmi R. (2004). Sputum CD34+IL-5Ralpha+ cells increase after allergen: Evidence for in situ eosinophilopoiesis. Am. J. Respir. Crit. Care Med..

[12] Sehmi R., Smith S.G., Kjarsgaard M., Radford K., Boulet L., Lemiere C., Prazma C.M., Ortega H., Martin J.G., Nair P. (2016). Role of local eosinophilopoietic processes in the development of airway eosinophilia in prednisone-dependent severe asthma. Clin. Exp. Allergy.

[13] Rodrigo-Muñoz J.M., Gil-Martínez M., Sastre B., Del Pozo V. (2021). Emerging Evidence for Pleiotropism of Eosinophils. Int. J. Mol. Sci..

[14] Rosenberg H.F., Phipps S., Foster P.S. (2007). Eosinophil trafficking in allergy and asthma. J. Allergy Clin. Immunol..

[15] Sanderson C.J. (1992). Interleukin-5, eosinophils, and disease. Blood.

[16] Mesnil C., Raulier S., Paulissen G., Xiao X., Birrell M.A., Pirottin D., Janss T., Starkl P., Ramery E., Henket M. (2016). Lung-resident eosinophils represent a distinct regulatory eosinophil subset. J. Clin. Investig..

[17] Tavernier J., Van der Heyden J., Verhee A., Brusselle G., Van Ostade X., Vandekerckhove J., North J., Rankin S.M., Kay A.B., Robinson D.S. (2000). Interleukin 5 regulates the isoform expression of its own receptor alpha-subunit. Blood.

[18] Youngblood B.A., Leung J., Falahati R., Williams J., Schanin J., Brock E.C., Singh B., Chang A.T., O’sullivan J.A., Schleimer R.P. (2020). Discovery, Function, and Therapeutic Targeting of Siglec-8. Cells.

[19] Wen T., Besse J.A., Mingler M.K., Fulkerson P.C., Rothenberg M.E. (2013). Eosinophil adoptive transfer system to directly evaluate pulmonary eosinophil trafficking in vivo. Proc. Natl. Acad. Sci. USA.

[20] Kanda A., Yun Y., Bui D.V., Nguyen L.M., Kobayashi Y., Suzuki K., Mitani A., Sawada S., Hamada S., Asako M. (2021). The multiple functions and subpopulations of eosinophils in tissues under steady-state and pathological conditions. Allergol. Int..

[21] Michail S., Mezoff E., Abernathy F. (2005). Role of selectins in the intestinal epithelial migration of eosinophils. Pediatr. Res..

[22] Gutierrez-Ramos J.C., Lloyd C., Gonzalo J.A. (1999). Eotaxin: From an eosinophilic chemokine to a major regulator of allergic reactions. Immunol. Today.

[23] Simson L., Foster P.S. (2000). Chemokine and cytokine cooperativity: Eosinophil migration in the asthmatic response. Immunol. Cell Biol..

[24] Sehmi R., Dorman S., Baatjes A., Watson R., Foley R., Ying S., Robinson D.S., Kay A.B., O'BYrne P.M., Denburg J.A. (2003). Allergen-induced fluctuation in CC chemokine receptor 3 expression on bone marrow CD34+ cells from asthmatic subjects: Significance for mobilization of haemopoietic progenitor cells in allergic inflammation. Immunology.

[25] Ravensberg A.J., Ricciardolo F.L., van Schadewijk A., Rabe K.F., Sterk P.J., Hiemstra P.S., Mauad T. (2005). Eotaxin-2 and eotaxin-3 expression is associated with persistent eosinophilic bronchial inflammation in patients with asthma after allergen challenge. J. Allergy Clin. Immunol..

[26] Jia G.Q., Gonzalo J.A., Hidalgo A., Wagner D., Cybulsky M., Gutierrez-Ramos J.C. (1999). Selective eosinophil transendothelial migration triggered by eotaxin via modulation of Mac-1/ICAM-1 and VLA-4/VCAM-1 interactions. Int. Immunol..

[27] Chakir J., Shannon J., Molet S., Fukakusa M., Elias J., Laviolette M., Boulet L.-P., Hamid Q. (2003). Airway remodeling-associated mediators in moderate to severe asthma: Effect of steroids on TGF-beta, IL-11, IL-17, and type I and type III collagen expression. J. Allergy Clin. Immunol..

[28] Gleich G.J. (2000). Mechanisms of eosinophil-associated inflammation. J. Allergy Clin. Immunol..

[29] Ueki S., Miyabe Y., Yamamoto Y., Fukuchi M., Hirokawa M., Spencer L.A., Weller P.F. (2019). Charcot-Leyden Crystals in Eosinophilic Inflammation: Active Cytolysis Leads to Crystal Formation. Curr. Allergy Asthma Rep..

[30] Fettrelet T., Gigon L., Karaulov A., Yousefi S., Simon H.U. (2021). The Enigma of Eosinophil Degranulation. Int. J. Mol. Sci..

[31] McBrien C.N., Menzies-Gow A. (2017). The Biology of Eosinophils and Their Role in Asthma. Front. Med..

[32] Ueki S., Tokunaga T., Melo R.C.N., Saito H., Honda K., Fukuchi M., Konno Y., Takeda M., Yamamoto Y., Hirokawa M. (2018). Charcot-Leyden crystal formation is closely associated with eosinophil extracellular trap cell death. Blood.

[33] Yousefi S., Gold J.A., Andina N., Lee J.J., Kelly A.M., Kozlowski E., Schmid I., Straumann A., Reichenbach J., Gleich G.J. (2008). Catapult-like release of mitochondrial DNA by eosinophils contributes to antibacterial defense. Nat. Med..

[34] Global Initiative for Asthma (2025). Global Strategy for Asthma Management and Prevention. http://www.ginasthma.org.

[35] Flood-Page P.T., Menzies-Gow A.N., Kay A.B., Robinson D.S. (2003). Eosinophil’s role remains uncertain as anti-interleukin-5 only partially depletes numbers in asthmatic airway. Am. J. Respir. Crit. Care Med..

[36] Shaker M., Briggs A., Dbouk A., Dutille E., Oppenheimer J., Greenhawt M. (2020). Estimation of Health and Economic Benefits of Clinic Versus Home Administration of Omalizumab and Mepolizumab. J. Allergy Clin. Immunol. Pract..

[37] Miyokawa R., Kivler C., Louie S., Godor D., Tan L., Kenyon N. (2020). Self-Administered Mepolizumab in the Management of Severe Asthma: Usability and Patient Acceptance. Patient Prefer. Adherence.

[38] Israel E., Canonica G.W., Brusselle G., Yang S., Howarth P.H., Martin A.L., Koufopoulou M., Smith S.G., Alfonso-Cristancho R. (2022). Real-life effectiveness of mepolizumab in severe asthma: A systematic literature review. J. Asthma.

[39] Sciurba F.C., Criner G.J., Christenson S.A., Martinez F.J., Papi A., Roche N., Bourbeau J., Korn S., Bafadhel M., Han M.K. (2025). Mepolizumab to Prevent Exacerbations of COPD with an Eosinophilic Phenotype. N. Engl. J. Med..

[40] Pilette C., Canonica G.W., Chaudhuri R., Chupp G., Lee F.E.-H., Lee J.K., Almonacid C., Welte T., Alfonso-Cristancho R., Jakes R.W. (2022). REALITI-A Study: Real-World Oral Corticosteroid-Sparing Effect of Mepolizumab in Severe Asthma. J. Allergy Clin. Immunol. Pract..

[41] Harrison T., Canonica G.W., Chupp G., Lee J., Schleich F., Welte T., Valero A., Gemzoe K., Maxwell A., Joksaite S. (2020). Real-world mepolizumab in the prospective severe asthma REALITI-A study: Initial analysis. Eur. Respir. J..

[42] Domvri K., Tsiouprou I., Bakakos P., Steiropoulos P., Katsoulis K., Antoniou K.M., Papaioannou A.I., Rovina N., Katsaounou P., Papamitsou T. (2025). Effect of mepolizumab in airway remodeling in patients with late-onset severe asthma with an eosinophilic phenotype. J. Allergy Clin. Immunol..

[43] Corren J., Weinstein S., Janka L., Zangrilli J., Garin M. (2016). Phase 3 Study of Reslizumab in Patients With Poorly Controlled Asthma: Effects Across a Broad Range of Eosinophil Counts. Chest.

[44] Kips J.C., O’Connor B.J., Langley S.J., Woodcock A., Kerstjens H.A.M., Postma D.S., Danzig M., Cuss F., Pauwels R.A. (2003). Effect of SCH55700, a humanized anti-human interleukin-5 antibody, in severe persistent asthma: A pilot study. Am. J. Respir. Crit. Care Med..

[45] Castro M., Zangrilli J., Wechsler M.E., Bateman E.D., Brusselle G.G., Bardin P., Murphy K., Maspero J.F., O'Brien C., Korn S. (2015). Reslizumab for inadequately controlled asthma with elevated blood eosinophil counts: Results from two multicentre, parallel, double-blind, randomised, placebo-controlled, phase 3 trials. Lancet Respir. Med..

[46] Bjermer L., Lemiere C., Maspero J., Weiss S., Zangrilli J., Germinaro M. (2016). Reslizumab for Inadequately Controlled Asthma With Elevated Blood Eosinophil Levels: A Randomized Phase 3 Study. Chest.

[47] Manka L.A., Guntur V.P., Denson J.L., Dunn R.M., Dollin Y.T., Strand M.J., Wechsler M.E. (2021). Efficacy and safety of reslizumab in the treatment of eosinophilic granulomatosis with polyangiitis. Ann. Allergy Asthma Immunol..

[48] Kolbeck R., Kozhich A., Koike M., Peng L., Andersson C.K., Damschroder M.M., Reed J.L., Woods R., Dall'Acqua W.W., Stephens G.L. (2010). MEDI-563, a humanized anti-IL-5 receptor alpha mAb with enhanced antibody-dependent cell-mediated cytotoxicity function. J. Allergy Clin. Immunol..

[49] Dagher R., Kumar V., Copenhaver A.M., Gallagher S., Ghaedi M., Boyd J., Newbold P., Humbles A.A., Kolbeck R. (2022). Novel mechanisms of action contributing to benralizumab’s potent anti-eosinophilic activity. Eur. Respir. J..

[50] Louis R., Harrison T.W., Chanez P., Menzella F., Philteos G., Cosio B.G., Lugogo N.L., de Luiz G., Burden A., Adlington T. (2023). Severe Asthma Standard-of-Care Background Medication Reduction With Benralizumab: ANDHI in Practice Substudy. J. Allergy Clin. Immunol. Pract..

[51] Bleecker E.R., FitzGerald J.M., Chanez P., Papi A., Weinstein S.F., Barker P., Sproule S., Gilmartin G., Aurivillius M., Werkström V. (2016). Efficacy and safety of benralizumab for patients with severe asthma uncontrolled with high-dosage inhaled corticosteroids and long-acting β_2_-agonists (SIROCCO): A randomised, multicentre, placebo-controlled phase 3 trial. Lancet.

[52] FitzGerald J.M., Bleecker E.R., Nair P., Korn S., Ohta K., Lommatzsch M., Ferguson G.T., Busse W.W., Barker P., Sproule S. (2016). Benralizumab, an anti-interleukin-5 receptor α monoclonal antibody, as add-on treatment for patients with severe, uncontrolled, eosinophilic asthma (CALIMA): A randomised, double-blind, placebo-controlled phase 3 trial. Lancet.

[53] Nair P., Wenzel S., Rabe K.F., Bourdin A., Lugogo N.L., Kuna P., Barker P., Sproule S., Ponnarambil S., Goldman M. (2017). Oral Glucocorticoid-Sparing Effect of Benralizumab in Severe Asthma. N. Engl. J. Med..

[54] Busse W.W., Bleecker E.R., FitzGerald J.M., Ferguson G.T., Barker P., Sproule S., Olsson R., Martin U.J., Goldman M., Yañez A. (2019). Long-term safety and efficacy of benralizumab in patients with severe, uncontrolled asthma: 1-year results from the BORA phase 3 extension trial. Lancet Respir. Med..

[55] Korn S., Bourdin A., Chupp G., Cosio B.G., Arbetter D., Shah M., Gil E.G. (2021). Integrated Safety and Efficacy Among Patients Receiving Benralizumab for Up to 5 Years. J. Allergy Clin. Immunol. Pract..

[56] Vultaggio A., Aliani M., Altieri E., Bracciale P., Brussino L., Caiaffa M.F., Cameli P., Canonica G.W., Caruso C., Centanni S. (2023). Long-term effectiveness of benralizumab in severe eosinophilic asthma patients treated for 96-weeks: Data from the ANANKE study. Respir. Res..

[57] Liu T., Wang F., Wang G., Mao H. (2018). Efficacy and safety of benralizumab in patients with eosinophilic asthma: A meta-analysis of randomized placebo-controlled trials. Front. Med..

[58] Jackson D.J., Wechsler M.E., Jackson D.J., Bernstein D., Korn S., Pfeffer P.E., Chen R., Saito J., de Luíz Martinez G., Dymek L. (2024). Twice-Yearly Depemokimab in Severe Asthma with an Eosinophilic Phenotype. N. Engl. J. Med..

[59] Wechsler M.E., Pavord I.D., Panettieri R.A., Buhl R., Kraft M., Rupani H., Schleich F., Jackson D.J., Muccino D., Forth R. (2026). Early and Sustained Efficacy of Depemokimab in Type 2 Asthma: A Pooled Analysis of the SWIFT-1/-2 Studies. J. Allergy Clin. Immunol. Pract..

[60] Piazzetta G.L., Lobello N., Di Agostino S., Coscarella I., Pelaia C., Di Vito A., Bria J., Filardo A., Aloisio A., Lupia C. (2026). Mucosal Remodeling in Chronic Rhinosinusitis with Nasal Polyps: The Role of Innate Lymphoid Cells and Reprogramming Under IL-4Rα Blockade. Int. J. Mol. Sci..

[61] Blauvelt A., de Bruin-Weller M., Gooderham M., Cather J.C., Weisman J., Pariser D., Simpson E.L., Papp K.A., Hong H.C.-H., Rubel D. (2017). Long-term management of moderate-to-severe atopic dermatitis with dupilumab and concomitant topical corticosteroids (LIBERTY AD CHRONOS): A 1-year, randomised, double-blinded, placebo-controlled, phase 3 trial. Lancet.

[62] (2021). Tezspire (Tezepelumab) US Prescribing Information. https://www.accessdata.fda.gov/drugsatfda_docs/label/2021/761224s000lbl.pdf.

[63] Ziegler S.F., Roan F., Bell B.D., Stoklasek T.A., Kitajima M., Han H. (2013). The biology of thymic stromal lymphopoietin (TSLP). Adv. Pharmacol..

[64] Tanaka J., Watanabe N., Kido M., Saga K., Akamatsu T., Nishio A., Chiba T. (2009). Human TSLP and TLR3 ligands promote differentiation of Th17 cells with a central memory phenotype under Th2-polarizing conditions. Clin. Exp. Allergy.

[65] Liu S., Verma M., Michalec L., Liu W., Sripada A., Rollins D., Good J., Ito Y., Chu H., Gorska M.M. (2018). Steroid resistance of airway type 2 innate lymphoid cells from patients with severe asthma: The role of thymic stromal lymphopoietin. J. Allergy Clin. Immunol..

[66] Menzies-Gow A., Colice G., Griffiths J.M., Almqvist G., Ponnarambil S., Kaur P., Ruberto G., Bowen K., Hellqvist Å., Mo M. (2020). NAVIGATOR: A phase 3 multicentre, randomized, double-blind, placebo-controlled, parallel-group trial to evaluate the efficacy and safety of tezepelumab in adults and adolescents with severe, uncontrolled asthma. Respir. Res..

[67] Corren J., Parnes J.R., Wang L., Mo M., Roseti S.L., Griffiths J.M., van der Merwe R. (2017). Tezepelumab in Adults with Uncontrolled Asthma. N. Engl. J. Med..

[68] Wechsler M.E., Menzies-Gow A., Brightling C.E., Kuna P., Korn S., Welte T., Griffiths J.M., Sałapa K., Hellqvist Å., Almqvist G. (2022). Evaluation of the oral corticosteroid-sparing effect of tezepelumab in adults with oral corticosteroid-dependent asthma (SOURCE): A randomised, placebo-controlled, phase 3 study. Lancet Respir. Med..

[69] Sverrild A., Hansen S., Hvidtfeldt M., Clausson C.-M., Cozzolino O., Cerps S., Uller L., Backer V., Erjefält J., Porsbjerg C. (2021). The effect of tezepelumab on airway hyperresponsiveness to mannitol in asthma (UPSTREAM). Eur. Respir. J..

[70] Menzies-Gow A., Wechsler M.E., Brightling C.E., Korn S., Corren J., Israel E., Chupp G., Bednarczyk A., Ponnarambil S., Caveney S. (2023). Long-term safety and efficacy of tezepelumab in people with severe, uncontrolled asthma (DESTINATION): A randomised, placebo-controlled extension study. Lancet Respir. Med..

[71] Pelaia C., Heffler E., Crimi C., Maglio A., Vatrella A., Pelaia G., Canonica G.W. (2022). Interleukins 4 and 13 in Asthma: Key Pathophysiologic Cytokines and Druggable Molecular Targets. Front. Pharmacol..

[72] Pelaia C., Paoletti G., Puggioni F., Racca F., Pelaia G., Canonica G.W., Heffler E. (2019). Interleukin-5 in the Pathophysiology of Severe Asthma. Front. Physiol..

[73] Younis U.S., Vallorz E., Addison K.J., Ledford J.G., Myrdal P.B. (2019). Preformulation and Evaluation of Tofacitinib as a Therapeutic Treatment for Asthma. AAPS PharmSciTech.

[74] Kudlacz E., Conklyn M., Andresen C., Whitney-Pickett C., Changelian P. (2008). The JAK-3 inhibitor CP-690550 is a potent anti-inflammatory agent in a murine model of pulmonary eosinophilia. Eur. J. Pharmacol..

[75] Nakamura Y., Ghaffar O., Olivenstein R., Taha R.A., Soussi-Gounni A., Zhang D.-H., Ray A., Hamid Q. (1999). Gene expression of the GATA-3 transcription factor is increased in atopic asthma. J. Allergy Clin. Immunol..

[76] Klein-Hessling S., Jha M.K., Santner-Nanan B., Berberich-Siebelt F., Baumruker T., Schimpl A., Serfling E. (2003). Protein kinase A regulates GATA-3-dependent activation of IL-5 gene expression in Th2 cells. J. Immunol..

[77] Krug N., Hohlfeld J.M., Kirsten A.M., Kornmann O., Beeh K.M., Kappeler D., Korn S., Ignatenko S., Timmer W., Rogon C. (2015). Allergen-induced asthmatic responses modified by a GATA3-specific DNAzyme. N. Engl. J. Med..

[78] Floyd H., Ni J., Cornish A.L., Zeng Z., Liu D., Carter K.C., Steel J., Crocker P.R. (2000). Siglec-8. A novel eosinophil-specific member of the immunoglobulin superfamily. J. Biol. Chem..

[79] Kikly K.K., Bochner B.S., Freeman S.D., Tan K., Gallagher K.T., D’aLessio K.J., Holmes S.D., Abrahamson J.A., Erickson-Miller C.L., Murdock P.R. (2000). Identification of SAF-2, a novel siglec expressed on eosinophils, mast cells, and basophils. J. Allergy Clin. Immunol..

[80] Kerr S.C., Gonzalez J.R., Schanin J., Peters M.C., Lambrecht B.N., Brock E.C., Charbit A., Ansel K.M., Youngblood B.A., Fahy J.V. (2020). An anti-siglec-8 antibody depletes sputum eosinophils from asthmatic subjects and inhibits lung mast cells. Clin. Exp. Allergy.

[81] Sulaiman I., Lim J.C., Soo H.L., Stanslas J. (2016). Molecularly targeted therapies for asthma: Current development, challenges and potential clinical translation. Pulm. Pharmacol. Ther..

[82] Pelaia C., Crimi C., Vatrella A., Busceti M.T., Gaudio A., Garofalo E., Bruni A., Terracciano R., Pelaia G. (2020). New treatments for asthma: From the pathogenic role of prostaglandin D_2_ to the therapeutic effects of fevipiprant. Pharmacol. Res..

[83] Cusack R.P., Whetstone C.E., Xie Y., Ranjbar M., Gauvreau G.M. (2021). Regulation of Eosinophilia in Asthma-New Therapeutic Approaches for Asthma Treatment. Cells.

[84] Gonem S., Berair R., Singapuri A., Hartley R., Laurencin M.F.M., Bacher G., Holzhauer B., Bourne M., Mistry V., Pavord I.D. (2016). Fevipiprant, a prostaglandin D2 receptor 2 antagonist, in patients with persistent eosinophilic asthma: A single-centre, randomised, double-blind, parallel-group, placebo-controlled trial. Lancet Respir. Med..

[85] Brightling C.E., Gaga M., Inoue H., Li J., Maspero J., Wenzel S., Maitra S., Lawrence D., Brockhaus F., Lehmann T. (2021). Effectiveness of fevipiprant in reducing exacerbations in patients with severe asthma (LUSTER-1 and LUSTER-2): Two phase 3 randomised controlled trials. Lancet Respir. Med..

[86] Barnes N., Pavord I., Chuchalin A., Bell J., Hunter M., Lewis T., Parker D., Payton M., Collins L.P., Pettipher R. (2012). A randomized, double-blind, placebo-controlled study of the CRTH2 antagonist OC000459 in moderate persistent asthma. Clin. Exp. Allergy.

[87] Combadiere C., Ahuja S.K., Murphy P.M. (1995). Cloning and functional expression of a human eosinophil CC chemokine receptor. J. Biol. Chem..

[88] Gauvreau G.M., FitzGerald J.M., Boulet L.P., Watson R.M., Hui L., Villineuve H., Scime T.X., Schlatman A.R., Obminski C., Kum J. (2018). The effects of a CCR3 inhibitor, AXP1275, on allergen-induced airway responses in adults with mild-to-moderate atopic asthma. Clin. Exp. Allergy.

[89] Neighbour H., Boulet L.P., Lemiere C., Sehmi R., Leigh R., Sousa A.R., Martin J., Dallow N., Gilbert J., Allen A. (2014). Safety and efficacy of an oral CCR3 antagonist in patients with asthma and eosinophilic bronchitis: A randomized, placebo-controlled clinical trial. Clin. Exp. Allergy.

[90] Gauvreau G.M., Boulet L.P., Cockcroft D.W., Baatjes A., Cote J., Deschesnes F., Davis B., Strinich T., Howie K., Duong M. (2008). Antisense therapy against CCR3 and the common beta chain attenuates allergen-induced eosinophilic responses. Am. J. Respir. Crit. Care Med..

[91] Gauvreau G.M., Pageau R., Séguin R., Carballo D., Gauthier J., D’aNjou H., Campbell H., Watson R., Mistry M., Parry-Billings M. (2011). Dose-response effects of TPI ASM8 in asthmatics after allergen. Allergy.

[92] Rogers L., Jesenak M., Bjermer L., Hanania N.A., Seys S.F., Diamant Z. (2023). Biologics in severe asthma: A pragmatic approach for choosing the right treatment for the right patient. Respir. Med..

[93] Miralles-López J.C., Antolín-Amérigo D., García-Moguel I., Domínguez-Ortega J., Delgado-Romero J., Quirce S. (2024). Positioning of Tezepelumab in Severe Asthma. J. Investig. Allergol. Clin. Immunol..

[94] Kavanagh J.E., Hearn A.P., Jackson D.J. (2021). A pragmatic guide to choosing biologic therapies in severe asthma. Breathe.

[95] Pepper A.N., Hanania N.A., Humbert M., Casale T.B. (2021). How to Assess Effectiveness of Biologics for Asthma and What Steps to Take When There Is Not Benefit. J. Allergy Clin. Immunol. Pract..

[96] Scioscia G., Nolasco S., Campisi R., Quarato C.M.I., Caruso C., Pelaia C., Portacci A., Crimi C. (2023). Switching Biological Therapies in Severe Asthma. Int. J. Mol. Sci..

[97] Menzies-Gow A.N., McBrien C., Unni B., Porsbjerg C.M., Al-Ahmad M., Ambrose C.S., Assing K.D., von Bülow A., Busby J., Cosio B.G. (2022). Real World Biologic Use and Switch Patterns in Severe Asthma: Data from the International Severe Asthma Registry and the US CHRONICLE Study. J. Asthma Allergy.

[98] Perez-de-Llano L., Scelo G., Tran T.N., Le T.T., Fagerås M., Cosio B.G., Peters M., Pfeffer P.E., Al-Ahmad M., Al-Lehebi R.O. (2024). Exploring Definitions and Predictors of Severe Asthma Clinical Remission after Biologic Treatment in Adults. Am. J. Respir. Crit. Care Med..

[99] Lupia C., Marchi G., Chiarella E., Piazzetta G.L., Lobello N., Crimi C., Poto R., Maglio A., Vatrella A., Pelaia G. (2026). Predictive Factors And Treatments Associated with Clinical Remission in Severe Eosinophilic Asthma. J. Inflamm. Res..

[100] Canonica G.W., Blasi F., Paggiaro P., Heffler E., Braido F., Brussino L., Scioscia G., Cardini C., Oriecuia C., Sala I. (2025). SANIstudy group Biologics as well as inhaled anti-asthmatic therapy achieve clinical remission: Evidence from the Severe Asthma Network in Italy (SANI). World Allergy Organ. J..

